# Issues in the design and conduct of a multicentre trial in UK ambulance services

**DOI:** 10.1186/1745-6215-14-S1-O82

**Published:** 2013-11-29

**Authors:** Simon Gates, Jessica Horton, Susie Hennings, Gavin Perkins

**Affiliations:** 1 Warwick Clinical Trials Unit, Coventry, UK

## 

Few trials have been conducted by UK ambulance services, and well-known examples of trials experiencing difficulties has led to the perception that prehospital care is a difficult environment for trials.

PARAMEDIC is an HTA-funded trial comparing mechanical chest compression (using the LUCAS-2 device) with manual chest compression, during attempted resuscitation after cardiac arrest. It is recruiting 4,344 patients from four ambulance services in the UK; the primary outcome is 30 day survival.

The trial uses cluster randomisation, with allocation by vehicles. All eligible cardiac arrests where a trial vehicle was first in attendance are included, which. This ensures minimal chance of differential recruitment between the LUCAS and manual chest compression arms, avoiding one of the main threats to cluster randomised trials.

The study has raised a number of interesting methodological challenges, including: 1. How to identify all cardiac arrests attended by trial vehicles from multiple imperfect data sources; 2. Movements of vehicles between ambulance stations; trial vehicles often move away from their base station to a non-participating station, and hence do not recruit to the trial; 3. Device management; devices are easily removed from vehicles, and also need to be located for servicing or product recalls; 4. Compliance; although the crews cannot decide not to recruit a patient, they can decide not to use the LUCAS device; there has been significant non-use of the devices, with important consequences for the analysis of the trial.

